# Temperature string device (TSD): A new low-cost instrument for sea ice observations in remote areas

**DOI:** 10.1016/j.ohx.2025.e00668

**Published:** 2025-07-02

**Authors:** Lasse Alexander Nissen Pedersen, Jeppe Don, Claus Melvad, Søren Rysgaard

**Affiliations:** a Department of Mechanical and Production Engineering, Aarhus University, Katrinebjergvej 89F, DK-8200 Aarhus N, Denmark; b Department of Biology, Arctic Research Centre & CIFAR at Aarhus University, Ole Worms Allé 1, DK-8000 Aarhus C, Denmark

**Keywords:** Arctic, Climate, Temperature measurement, Sea ice thickness, Remote deployment, Low-cost

## Abstract

Warmer conditions in the Arctic regions are causing sea ice to melt. Sea ice plays critical roles in reflecting solar radiation, mitigating heat absorption and slowing temperature rise. However, the reduction in annual sea ice formation, driven by rising temperatures, contributes to a positive feedback loop that accelerates further warming. This underscores the importance of monitoring seasonal sea ice growth. To address this need, a Temperature String Device has been developed. This innovative, low-cost, compact, and customizable solution is designed for easy deployment, requiring just one person or even a robot for non-human deployment. The device measures temperature throughout the sea ice and transmits data in real-time to the cloud, enabling immediate analysis to estimate sea ice thickness. Its default configuration collects 30 data points over a 3-meter depth at 30-minute intervals and remains operational for up to one year. Its affordability allows production of multiple units, enabling widespread deployment and enhancing spatial resolution. Furthermore, its suitability for robotic deployments makes it ideal for remote, inaccessible locations, facilitating simultaneous monitoring.

## Graphical abstract

**Figure:**
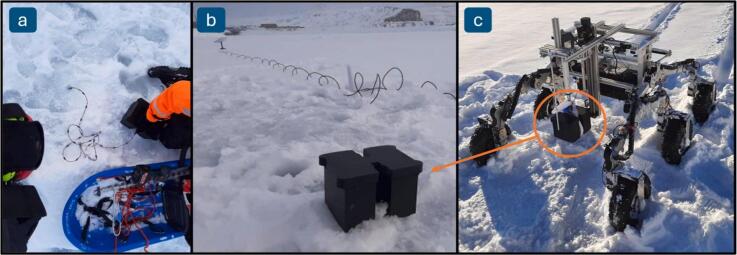

Specifications table

**Table:** 

Hardware name	Temperature String Device
Subject area	Engineering and materials science
Hardware type	Field measurements and sensors
Closest commercial analog	- Snow Ice Mass Balance Apparatus (SIMBA) − SAMS enterprise - Digital Temperature Cable + F605 Data Logger − beadedstream
Open source license	CC BY 4.0
Cost of hardware	475 USD
Source file repository	https://doi.org/10.17632/2pxhn7f35n.2

## 1 Hardware in context

The Earth's temperature is increasing at an alarming rate [1], causing significant environmental changes. These include rising sea levels, disruptions to ocean currents, more extreme weather events, and the accelerated melting of sea ice [2]. One of the most vulnerable regions is the Arctic [3]. A key factor driving higher temperature in the Arctic is the reduction of sea ice, which decreases the amount of solar radiation reflected into space [4]. Consequently, the extent and thickness of sea ice are critical not only for the Arctic environment but also for the stability of the global climate system.

Satellites are highly effective at measuring the extent of sea ice but face challenges in accurately determining its thickness. Additionally, they struggle to measure conditions within and beneath the sea ice—such as light levels, temperature, and salinity. Recently, the CryoSat and ICESat-2 satellites were launched to improve the measurement of sea ice thickness [5]. However, ground-truth measurements are essential for calibrating and validating satellite data.

Commercial solution offered by, for instance, SAMS enterprise [6] and beadedstream are available. Compared to TSD these systems are more expensive. This is partially due to these systems also including other sea ice parameters and the fact that these commercial products need to gain a profit as well. In addition, an advantage of the TSD is its small size and flexible TS for easier transportation and deployment, as well as its customizability regarding length and sensor spacing.

**Fig. 1:**
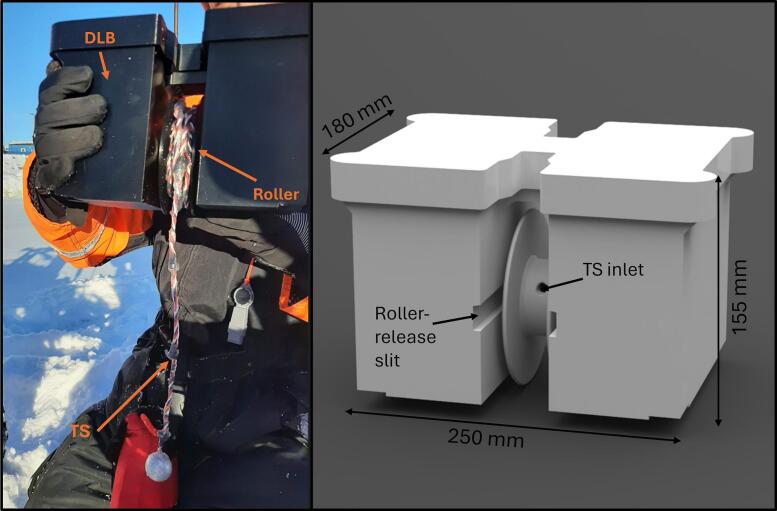
TSD and some of its components. Left: DLB with attached TS. Right: DLB cad with roller-release mechanism attached.

The TSD (Fig. 1) measures the temperature of sea ice and transmits the data to the cloud via satellite or internet connection. The TSD comprises two main components: the Temperature String (TS) and the Data Logging Box (DLB). The TS is deployed into a drilled hole in the sea ice, allowing the temperature conditions and the growth of sea ice to be monitored by analyzing the recorded temperatures, providing the local freezing point of the sea ice is known (the depth where the temperature reaches the freezing point of seawater; approximately −1.8 °C, depending on salinity). The string is connected to the DLB, which collects and uploads the temperature data while housing all necessary electronics.

The DLB is positioned on top of the drilled sea ice hole after the TS is inserted. The TSD is designed for autonomous, robotic deployment, eliminating the need for human interaction. This feature enables its use in locations that are often inaccessible to humans. The device's design is compact and user-friendly, making it highly manageable. Additionally, the TSD is cost-effective compared to existing commercial products that provide similar results, further enhancing its practicality and appeal.

## 2 Hardware description

The TSD described here is a cost-effective, easy-to-use, and versatile temperature sensing solution. The TS is composed of DS18B20 [7] temperature sensors. In its default configuration (described in this paper), the TSD includes 30 temperature sensors evenly spaced at 10 cm intervals along a 3-meters long wire. The minimum spacing between sensors depend on the manufacturers soldering skill as it relies on the length of the wires in between. For production of the TS, the main methods include 3D printing, soldering and coating. Key advantages of the TSD concept include:

• Cost-effective
• Ease of setup
• Flexible string
• Battery for > 1 year of data gathering
• Adjustable resolution (length between sensors)
• Portable, versatile and compact

### 2.1 Temperature string

**Fig. 2:**
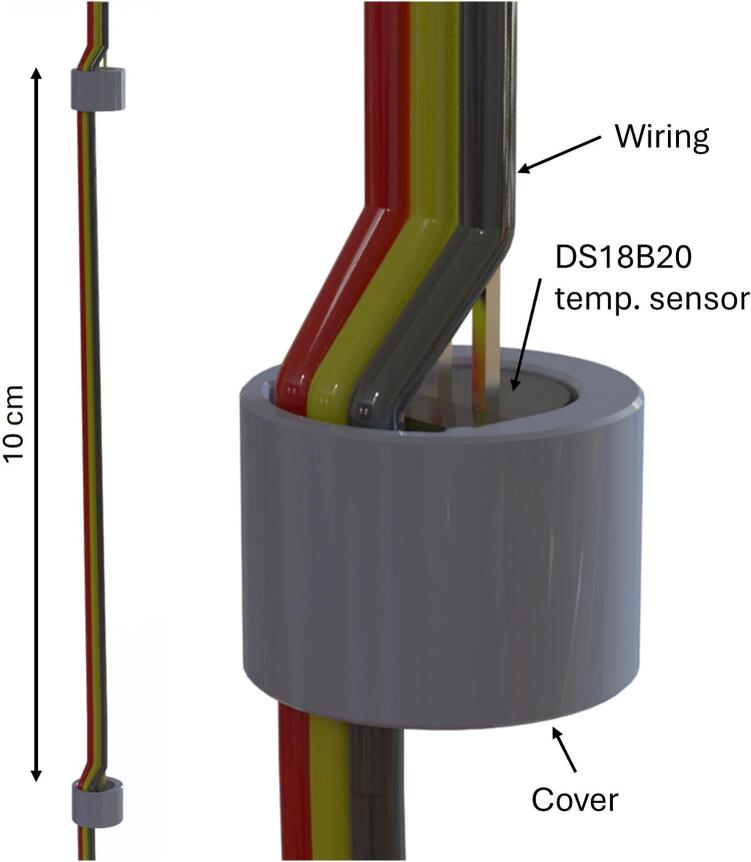
TS assembly before coating.

The TS is made of 30 DS18B20 sensors, resulting in a 3-meter-long string (Fig. 2). The DS18B20 sensor is chosen for its robust 1-wire technology, where each sensor has a unique 64-bit serial code, allowing nearly unlimited sensors to function on the same bus. This ensures consistent wire management and ease of expansion. The sensor’s thermal resolution is 12 bits, corresponding to a temperature resolution of 0.0625°C. [7].

For waterproofing and corrosive protection, the string is covered in conformal coating (Dow Dowsil 3140 RTV [8]). That is made of a one-component silicone which cures to a flexible and elastic silicone rubber by reaction with atmospheric moisture at room temperature. A simple ball sinker is attached at the end to ensure the string remains submerged vertically. The TSD is calibrated in a LAUDA bath to ensure high accuracy across its operational temperature range. The calibrations certificate is included in the repository (Table 1).

### 2.2 Data logging box

The TS is attached to a data logging box, which contains batteries and a PCB with electronics (Fig. 1). The main parts are 3D printed in PETG. The DLB is made with the purpose of being deployable by a remote-controlled robot: It is symmetrical for an optimal mass distribution and there is an attaching slit for a TS roller-release mechanism. An article detailing the remote-controlled robot is currently in development. The box features 4 structural walls that secures the batteries and adds fixation points for the PCB. Furthermore, it secures room for the TS to protect it from the batteries. There are attachment indentations with threaded inserts that allows the possibility to attach legs to raise the DLB from the ground. This possibility has not been used in the executed missions.

The TS is attached to the DLB through a hole in a hollow roller (Fig. 1). The rest of the TS is rolled around the roller and can be transported ready to be released. The roller rolls on two bearings in each side.

### 2.3 Electronics

**Fig. 3:**
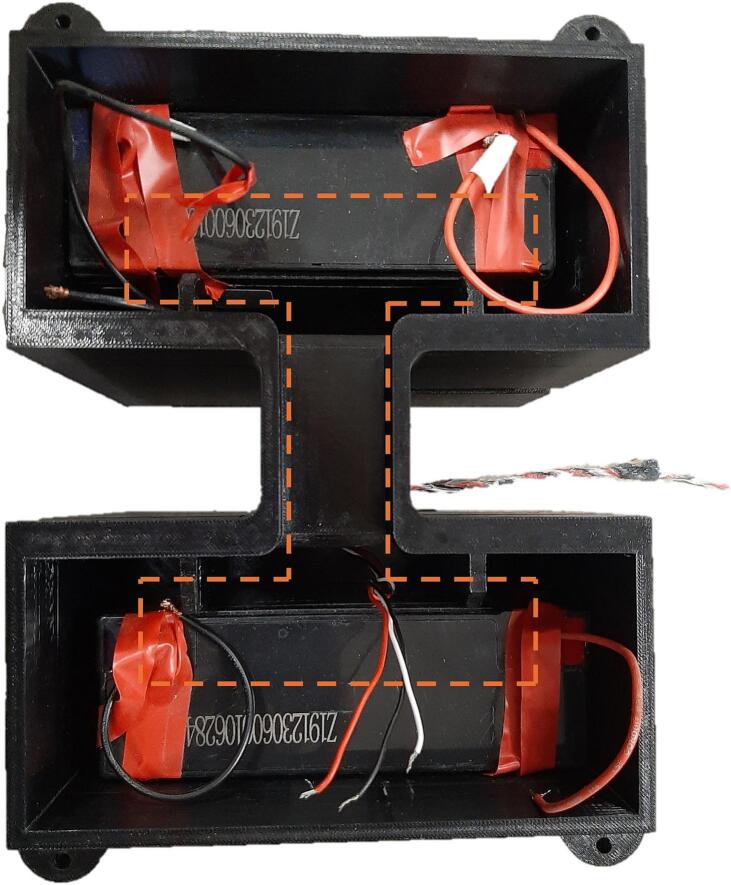
DLB from the inside containing only the batteries and with the TS entered. The dashed lines represent the placement of the PCB.

**Fig. 4:**
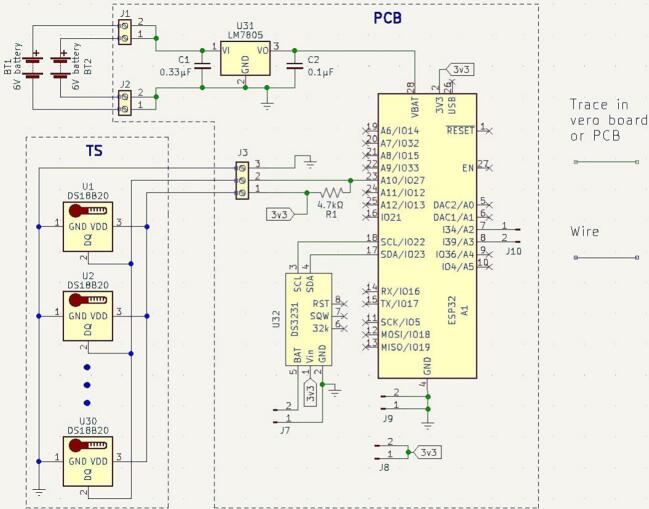
Circuit diagram of TSD.

The electronics in the DLB consist of a PCB, an ESP32 Feather microcontroller, two lead-crystal batteries as well as the attachment of the TS. The inside of the DLB only with batteries and the TS can be observed in Fig. 3. The overall electronics diagram can be observed in Fig. 4.

For powering the TSD, lead-crystal batteries are used. This battery type is efficient at very low temperatures and self-discharges very slowly. The two batteries will be able to supply the entire TSD for potentially 1 year of operation.

The microcontroller attached to the PCB is an ESP32 Feather board since it operates at −40 °C, it is low-powered with its possible sleep-modes, and it can connect to the cloud using Wi-Fi via an external internet access (e.g., an internet connection via satellite). With an external antenna the microcontroller can extend its range to the internet access point.

A voltage regulator surrounded by two capacitors converts the battery power into a stable 5 V power source to supply the microcontroller. The PCB also fastens the TS wires to it, and it connects the 1-wire communication between the TS and the microcontroller.

Furthermore, the PCB is equipped with a slot for a real time clock (RTC) if the user desires an increased accuracy of timing. Lastly, the PCB contains pin connections from the microcontroller for potential debugging and expanding possibilities for new features.

### 2.4 Software

**Fig. 5:**
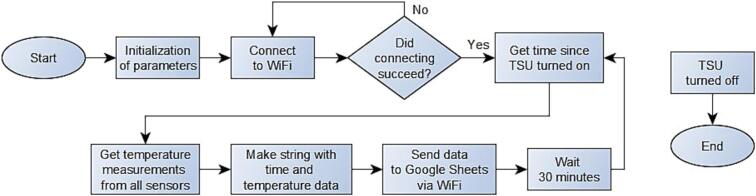
Software diagram for TSD. In the laboratory experiment, 2 min of waiting time was used instead of 30 min.

The software diagram of the TSD can be observed on Fig. 5.

**Fig. 6:**
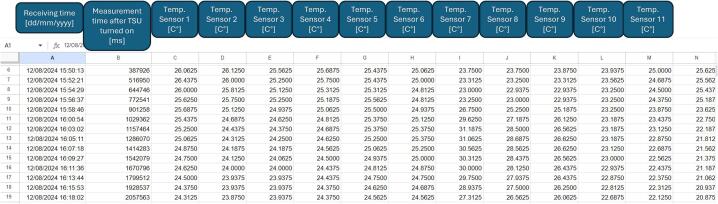
Example view of the Google Sheet file. There are sensor values for all 30 sensors.

The code starts connecting to the WiFi and only continues when the connection is successful. Hereafter, it reads the passed by time since the TSD was turned on, and then the temperature measurements for all sensors are read. The time for the temperature measurements and the temperature data are concatenated into a string, that is sent via WiFi to a Google Sheets file via a script set up for this file. This gets a string of current time stamp and the temperature measurements, then divides it into separate values as well as the time for receiving the string. These data will be passed into the respective cells in the Google Sheets file. An example of a Google Sheets file can be observed on Fig. 6.

### 2.5 Calibration

The TSD was calibrated against a reference PT-100 thermistor in a thermostatically controlled liquid (Cooling fluid) bath (LAUDA). The calibration was 2-way, performed at four temperatures. Following the calibration, the average, absolute residual deviation was found to $0.02^{{}^{\circ}}C$, with a maximum deviation of $0.10^{{}^{\circ}}C$. The calculated uncertainty budget resulted in an expanded standard uncertainty of ±0.0768 corresponding to a confidence level of approximately 95 %, which is consistent with other, previously reported results [9,10]. Further, elaborated details of the calibration and uncertainty budget, can be found in Mendeley Data repository.

### 2.6 Commercial comparison of competition

There are a few commercial products similar to the TSD. One is the Snow Ice Mass Balance Apparatus (SIMBA) developed by SAMS Enterprise. Another is the Digital Temperature Cable (DTC) and a compatible data logger (D605) developed by beadedstream.

A difference between the TSD and the similar products is the required setup facilities, where the TSD is deployable with just a robot and thereby no human presence, where the similar products require 1–2 people. Another difference is the compactness. Here the TSD has a packing volume of approximately 20x25x25 cm, which is way smaller than the alternatives.

**Fig. 7:**
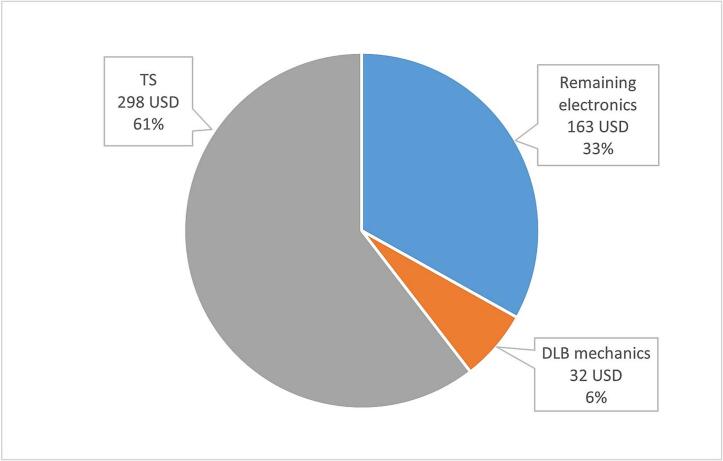
Diagram describing the cost distribution for TSD.

The SIMBA has a price of 8011 USD (6085 GBP) if only including the similar services as the TSD provides. However, the TSD is very adjustable compared to the SIMBA. The SIMBA has a limited number of commercialized configurations of length, spacing, and number of sensors, and therefore the TS has an advantage with its adjustability. Like the TS, the DTC by beadedstream also has prioritized flexibility in terms of cable length, spacing and number of sensors, however, the price difference for the DTC and the data logger is 8520 USD, and thereby, the cost of the TSD is lower. Be aware that a commercial price and a cost are compared in this case. The cost distribution for the TSD can be observed in Fig. 7.

### 2.7 Applications for TSD in general terms

- The TSD is a deployable device that can collect temperature data on a temperature string and thereby estimate the sea ice thickness from freezing temperature.
- It is cost-efficient enabling production of multiple devices to be deployed.
- It is compact and is designed to be deployed by either a person or a robot enabling deployments in remote and humanly inaccessible location.
- Temperature data is uploaded to the cloud for real-time analysis.
- As the TS is customizable, any specific configuration can easily be produced.
- In principle, the TS can be constructed for a length of hundreds of meters, however, with a maximum of 200 sensors.

## 3 Design files summary

**Table 1:** Design file summary.

**Design file name**	**File type**	**Open source license**	**Location of the file**
**CAD**			
001_DLB_with_roller	SLDASM-file	CC BY 4.0	https://doi.org/10.17632/2pxhn7f35n.2
101_Box	SLDPRT-file	CC BY 4.0	https://doi.org/10.17632/2pxhn7f35n.2
102_Lid	SLDPRT-file	CC BY 4.0	https://doi.org/10.17632/2pxhn7f35n.2
103_M4_threaded_insert	SLDPRT-file	CC BY 4.0	https://doi.org/10.17632/2pxhn7f35n.2
104_socket_countersunk_head_screw_din	SLDPRT-file	CC BY 4.0	https://doi.org/10.17632/2pxhn7f35n.2
110_Roller	SLDASM-file	CC BY 4.0	https://doi.org/10.17632/2pxhn7f35n.2
111_RollerShaftR	SLDPRT-file	CC BY 4.0	https://doi.org/10.17632/2pxhn7f35n.2
112_RollerShaftL	SLDPRT-file	CC BY 4.0	https://doi.org/10.17632/2pxhn7f35n.2
113_TS_Holder	SLDPRT-file	CC BY 4.0	https://doi.org/10.17632/2pxhn7f35n.2
114_radial_ball_bearing_68_din	SLDPRT-file	CC BY 4.0	https://doi.org/10.17632/2pxhn7f35n.2
002_DS18B20_Connection	SLDASM-file	CC BY 4.0	https://doi.org/10.17632/2pxhn7f35n.2
201_DS18B20_with_wires	SLDPRT-file	CC BY 4.0	https://doi.org/10.17632/2pxhn7f35n.2
202_DS18B20_Cover	SLDPRT-file	CC BY 4.0	https://doi.org/10.17632/2pxhn7f35n.2
**3D printing**			
301_Box_3D	3MF-file	CC BY 4.0	https://doi.org/10.17632/2pxhn7f35n.2
302_Lid_3D	3MF-file	CC BY 4.0	https://doi.org/10.17632/2pxhn7f35n.2
303_RollerShaftR_3D	3MF-file	CC BY 4.0	https://doi.org/10.17632/2pxhn7f35n.2
304_RollerShaftL_3D	3MF-file	CC BY 4.0	https://doi.org/10.17632/2pxhn7f35n.2
305_TS_Holder_3D	3MF-file	CC BY 4.0	https://doi.org/10.17632/2pxhn7f35n.2
306_DS18B20_Cover_3D	3MF-file	CC BY 4.0	https://doi.org/10.17632/2pxhn7f35n.2
**Electronics**			
203_DS18B20	SLDPRT-file	CC BY 4.0	https://doi.org/10.17632/2pxhn7f35n.2
PCB_TSD	Kicad file	CC BY 4.0	https://doi.org/10.17632/2pxhn7f35n.2
PCB_TSD_drill	Drill-file	CC BY 4.0	https://doi.org/10.17632/2pxhn7f35n.2
PCB Gerber files	Folder with Gerber files	CC BY 4.0	https://doi.org/10.17632/2pxhn7f35n.2
**Software**			
DataAcq_TS	Arduino code	CC BY 4.0	https://doi.org/10.17632/2pxhn7f35n.2
FindAddress_TS	Arduino code	CC BY 4.0	https://doi.org/10.17632/2pxhn7f35n.2
PrintTemp_TS	Arduino code	CC BY 4.0	https://doi.org/10.17632/2pxhn7f35n.2
Temperature_log_from_TSU_sheets_script	Word document containing JavaScript code	CC BY 4.0	https://doi.org/10.17632/2pxhn7f35n.2
**Calibration**			
Calibration Overview	XLSX-file	CC BY 4.0	https://doi.org/10.17632/2pxhn7f35n.2

## 4 Bill of materials summary

**Table 2:** Bill of materials.

**Designator**	**Component**	**Number**	**Cost per unit −currency**	**Total cost −** **currency**	**Source of materials**	**Material type**
**DLB**						
301_Box_3D	DLB box	1	14.25 USD	14.25 USD	https://3deksperten.dk/products/3de-basic-petg-true-cold-white-1–75-1 kg	Polymer
302_Lid_3D	DLB Lid	1	5.40 USD	5.40 USD	https://3deksperten.dk/products/3de-basic-petg-true-cold-white-1–75-1 kg	Polymer
303_RollerShaftR_3D	Roller Shaft Right	1	0.2 USD	0.2 USD	https://3deksperten.dk/products/3de-basic-petg-true-cold-white-1–75-1 kg	Polymer
304_RollerShaftL_3D	Roller Shaft Left	1	0.2 USD	0.2 USD	https://3deksperten.dk/products/3de-basic-petg-true-cold-white-1–75-1 kg	Polymer
305_TS_Holder_3D	TS Holder	1	1.10 USD	1.10 USD	https://3deksperten.dk/products/3de-basic-petg-true-cold-white-1–75-1 kg	Polymer
114_radial_ball_bearing_68_din	Bearings	2	4.05 USD	8.10 USD	https://www.mmaction.dk/6004rs-kugleleje.html	Metal
103_M4_threaded_insert	Threaded inserts (M4)	4	0.41 USD	1.64 USD	https://dk.rs-online.com/web/p/gevindindsatse/0278556	Metal
104_socket_countersunk_head_screw_din	Bolts (M4)	4	0.23 USD	0.92 USD	https://www.biltema.dk/byggeri/befastelser/maskinskruer/maskinskrue-forsanket-m4-x-20-mm-a4-25-stk-2000061046	Metal
**Temperature String**						
203_DS18B20	Temperature sensor	30	5.95 USD	178.50 USD	https://www.digikey.dk/da/products/detail/analog-devices-inc.-maxim-integrated/DS18B20%2BPAR/1197285	Semi-con1ductor
306_DS18B20_Cover_3D	Sensor Cover	30	0.10 USD	3.00 USD	https://3deksperten.dk/products/3de-basic-petg-true-cold-white-1–75-1 kg	Polymer
TS001	Conformal coating − Dow Dowsil 3140 RTV	1	109.66 USD	109.66 USD	https://www.diatom.dk/kemi/coating/silikone-coating/dow-dowsil-3140-rtv-310-ml-patron/	Inorganic
TS002	Wires (3.1 m)	3	0.96 USD	2.88 USD	https://dk.rs-online.com/web/p/monteringsledning/2882010	Metal
TS003	Weight	1	4.45 USD	4.45 USD	https://www.topgrej.dk/ifish-kuglelod-blyfri.html	Metal
**Remaining electronics**						
E001	Battery Lead-Crystal 6 V 12Ah	2	57.02 USD	114.04 USD	https://dk.rs-online.com/web/p/blybatterier/1934663	Other
A1	Adafruit HUZZAH32 − ESP32 Feather board	1	20.95 USD	20.95 USD	https://www.adafruit.com/product/3591	Non-specific
E003	PCB	1	4.51 USD	4.51 USD	https://www.pcbway.com/	Non-specific
U31	Voltage regulator −TO-220 L7805	1	0.29 USD	0.29 USD	https://ardustore.dk/produkt/voltage-regulator-5v-l7805	Non-specific
C2	Ceramic capacitor 0.1 μF	1	0.63 USD	0.63 USD	https://dk.farnell.com/en-DK/multicomp-pro/mccc50v223ky5p/ceramic-capacitor-0-022uf-50v/dp/1600821	Ceramic
C1	Ceramic capacitor 0.33μF	1	0.28 USD	0.28 USD	https://dk.farnell.com/en-DK/multicomp/mc0805b334k500a5-08 mm/cap-0–33-f-50v-10-x7r/dp/2309021	Ceramic
J1 / J2	Screw terminal 1x2	2	0.72 USD	1.44 USD	https://dk.rs-online.com/web/p/printklemraekker/8267226	Non-specific
J3	Screw terminal 1x3	1	1.15 USD	1.15 USD	https://dk.rs-online.com/web/p/printklemraekker/8267220	Non-specific
E012	Wire from battery (0.10 m)	4	0.10 USD	0.20 USD	https://dk.rs-online.com/web/p/monteringsledning/1964318	Metal
E014	Zip ties 2.5 mm	2	0.01 USD	0.02 USD	https://plakatstrips.dk/product/sorte-sidestrips-kabelbindere-200-x-25-mm-100-stk/	Polymer
R1	Resistance 4.7 kΩ	1	1.27 USD	1.27 USD	https://www.digikey.dk/da/products/detail/ohmite/MRA0207-100R-B-15PPM-TA/16369206	
U32	Optional: RTC DS3231	(1)	(17,65 USD)	(17,65 USD)	https://www.mouser.dk/ProductDetail/Adafruit/3013?qs = qrYS%2FWt5A16Mfm4Z0v0kNg%3D%3D	Non-specific

**Total cost:** 475.08 USD.

## 5 Build instructions

There are multiple parts that can be built independently of each other. These sub-assemblies will be described first, and eventually the final assembly will be described.

General considerations throughout the assembly include:

- 3D printed parts have been printed on consumer grade FDM printers. All 3D-printed parts must be checked for defects post printing and support material must be removed if used.
- When soldering, use proper ventilation.

For people with engineering or practical background, the building time in active man hours has been estimated to approximately 10 h in total: 9 h for building the TS, 25 min for completing the PCB, 10 min for 3D-printing and inserting threads, and finally 25 min for assembling all remaining parts.

### 5.1 DLB mechanical parts

Prepare mechanical parts for assembly.

**Fig. 8:**
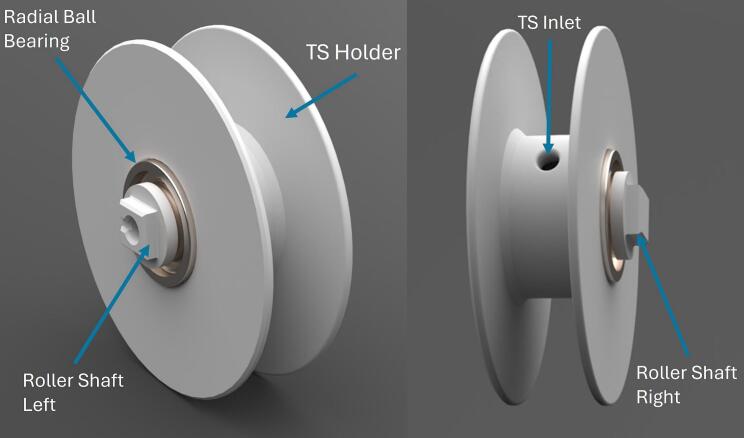
Assembled Roller with TS Holder, Roller Shafts and Radial Ball Bearing.

1. 3D-print TSD box in PETG using file “301_Box_3D”.
2. 3D-print TSD Lid in PETG using file “302_Lid_3D”.
3. Insert the threaded inserts (103_M4_threaded_insert) into the holes of the lid using a solder iron on the inserts, so the PETG will melt due to the thermal conducting between the PETG and the inserts.
4. 3D-print the Roller Shaft Right in PETG using file “303_RollerShaftR_3D”
5. 3D-print the Roller Shaft Left in PETG using file “303_RollerShaftL_3D”
6. 3D-print the TS Holder in PETG using file “305_TS_Holder_3D”
7. Put the two bearings (114_radial_ball_bearing_68_din) into each of the large holes in the TS Holder (Fig. 8).

### 5.2 TS Build

**Fig. 9:**
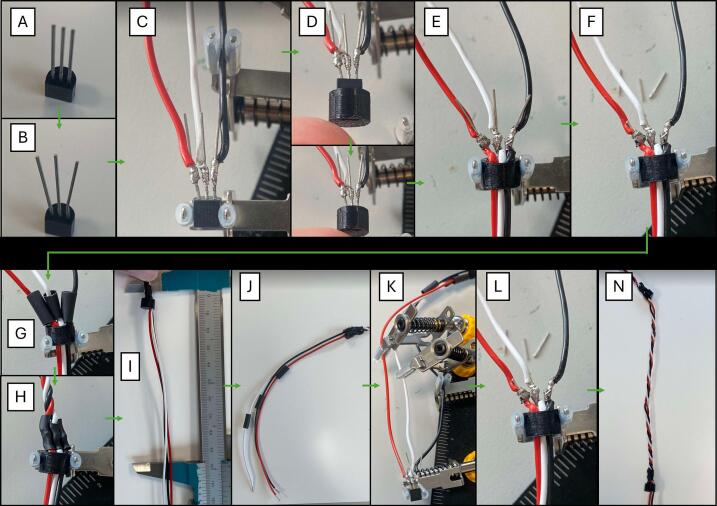
Build instructions for TS.

The assembly of the TS is divided into the first sensor and the rest of the sensors. Throughout the assembly of the TS, Fig. 9 will be referred to.

1. 3D-print 30 Sensor covers in PETG using file “306_DS18B20_Cover_3D”

First sensor:

1.1. Slightly open the DS18B20 legs making them ready for soldering [A→B]
1.2. Solder top wires to sensor legs (2–3 mm over sensor head) [C]
  a. Red to VCC
  b. Yellow/white to DQ
  c. Black to GND
1.3. Attach 3D printed cover [D]
1.4. Insert bottom wires through the hole in the cover [E]
1.5. Solder: [E]
  a. Red bottom wire to prior soldering at VCC
  b. Yellow/white bottom wire to prior soldering at DQ
  c. Black bottom wire to prior soldering at GND
1.6. Remove (cut) the excess part of the legs [F]
1.7. Slide heat shrink tube around each of the top wires and place it over the unified (sensor leg, top- and bottom wires) soldering [G]
1.8. Apply heat and shrink to fit [H]

Second to last sensor:

2.1. Use above sensors bottom wires as top wires
  a. Cut them to the correct length [I]
2.2. Before attaching to new sensor: [J]
  a. Prepare and place heat-shrink tubing on each of the top wires
2.3. Solder: [K]
  a. Red to VCC
  b. Yellow/white to DQ
  c. Black to GND
2.4. Attach 3D printed cover
2.5. Repeat 1.4. − 1.8 [L + N]
2.6. Optional: Test if assembled correctly by checking measurements (see points 14 to 15 in complete assembly)
2.7. Repeat until desired number of sensors is attached

### 5.3 TS coating

After having a finished TS Build, complete the following steps to coat it (use an area with a height of minimum the length of the TS):

1. Fill small container (with enough room for TS) with conformal coating
2. Submerge TS into container
3. From top end − slowly (1 cm/s) remove TS from container
4. Hang the TS over ground and allow it to cure for 24–48 h.
5. Check coating
  a. Add additional coating if needed
6. Optional: We have had success with double coating.
  a. Repeat process

### 5.4 PCB

**Fig. 10:**
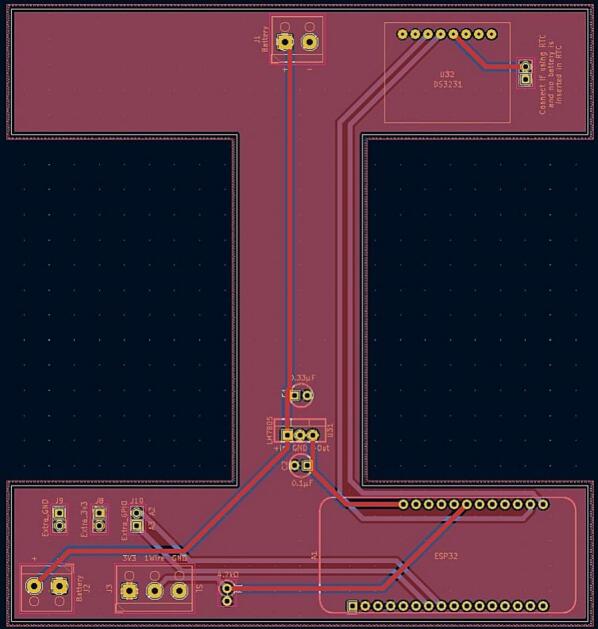
PCB layout for TSD.

1) Produce a PCB according to the files in the folder “PCB” in Table 1.
2) Solder the components to the PCB according to the designations in Table 2.
3) Optional: Solder the RTC to the PCB. See (Fig. 10).

### 5.5 Assembling everything

1) Create a Google Sheets file and setup an app script for this file. Insert the JavaScript code from file”Temperature_log_from_TSU_sheets_script” (Table 1) into the script. In this code insert the Google Sheets file ID as well as the sheet name for the desired sheet for the logged data to be input.
2) Strip both ends of 2 wires of approx. 25 cm, and 2 more wires of approx. 15 cm.
3) Attach the long wires to one battery and the short wires to another battery. Optionally, the wires can be soldered to the batteries for higher attachment with caution.
4) Insert the TS into the TS inlet (Fig. 8) of the hollow TS holder.
5) Insert the “Roller Shaft Right” and the “Roller Shaft Left” in on each of the bearings in the TS holder.
6) Pull, the un-coated wire ends of the TS through the hole of the “Roller Shaft Left”, and through the hole of the DLB box in the outside slit/Roller-release slit (Fig. 1). Pull the TS until it reaches the DLB box top (Fig. 3).
7) Insert the TS holder in the outside slit of the DLB box such the “Roller Shaft Left” matches the outside slit/Roller-release slit with the hole.
8) Wind up the TS on the roller, until the weight reaches the roller.
9) Insert the two batteries into the battery slot facing the poles towards the top of the DLB.
10) Put the PCB into the DLB so that is fits in the middle slit (Fig. 3), and such the microcontroller is placed towards the TS, which is to the right on Fig. 3.
11) Mount the TS wires to the component J3 in order specified on the PCB.
12) Fix the PCB by mounting it to the DLB using two zip ties through the holes in the DLB ribs and around across the PCB.
13) Upload the Arduino code from file”FindAddress_TS” (Table 1) to the microcontroller in Arduino IDE using the settings listed below. The code will print out the addresses for the 1-Wire communication of the temperature sensors. Note down the addresses.
  a. Board: Adafruit ESP32 Feather
  b. Upload speed: “921600”
  c. CPU Frequency: “240 MHz (WiFi/BT)”
  d. Partition Scheme: “Default 4 MB with spiffs (1.2 MB APP/1.5 MB SPIFFS)”
  e. Core Debug Level: “None”
  f. Arduino Runs On: “Core 1”
  g. Events Run On: “Core 1”
  h. Erase All Flash Before Sketch Upload: “Disabled”
  i. Port: <Choose the connected port for the microcontroller>
14) Insert the noted temperature sensor addresses into the Arduino code from the file”PrintTemp_TS”.

Upload the code to the microcontroller in Arduino IDE using the same settings as listed above. This code will print out the temperatures read by the temperature sensors. Identify the corresponding addresses to the respective temperature sensors.

15) In the Arduino code from the file”DataAcq_TS” (Table 1), insert the following information:
  a. Sorted temperature sensor addresses such the address for the first sensor corresponds to sensor 1 in the code, and so forth with the remaining temperature sensor addresses.
  b. WiFi name and password of the desired WiFi network for the microcontroller in the code.
  c. The Google sheets ID for the desired file for data logging.
  d. Optionally, the time interval between each measurement can be adjusted.
16) Upload the code to the microcontroller using the same settings as listed above.

## 6 Operation instructions

1) Ensure that the desired WiFi internet connection is available.
2) Mount the batteries to component J1 and J2, respectively, and according to the poles specified on the PCB. The microcontroller will automatically turn on and begin data gathering and transmission hereafter.
3) Put the lid on the TSD and mount it with the 4 screws near each corner.
4) At the desired deployment location, drill a hole through the sea ice.
5) Unwind the TS into the hole of the ice.
6) Leave the TSD on top of the hole.
7) Monitor the data logging in real time in the Google Sheets file.
8) Data in Google Sheets can be downloaded whenever data is needed.

## 7 Validation and characterization

### 7.1 Functional test in Nuuk

**Fig. 11:**
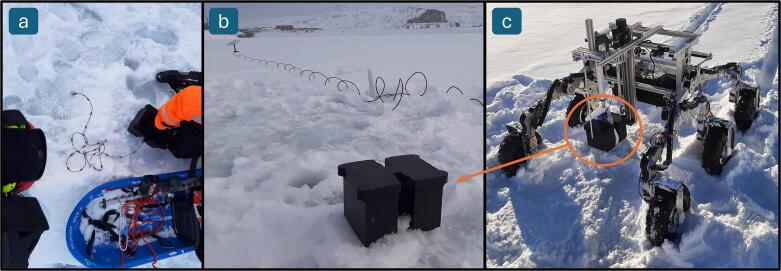
Test setup in Nuuk: a) TS on top of sea ice, b) TSD with TS inserted in ice and with internet connection through satellite in background and c) TSD on rover ready for deployment.

**Fig. 12:**
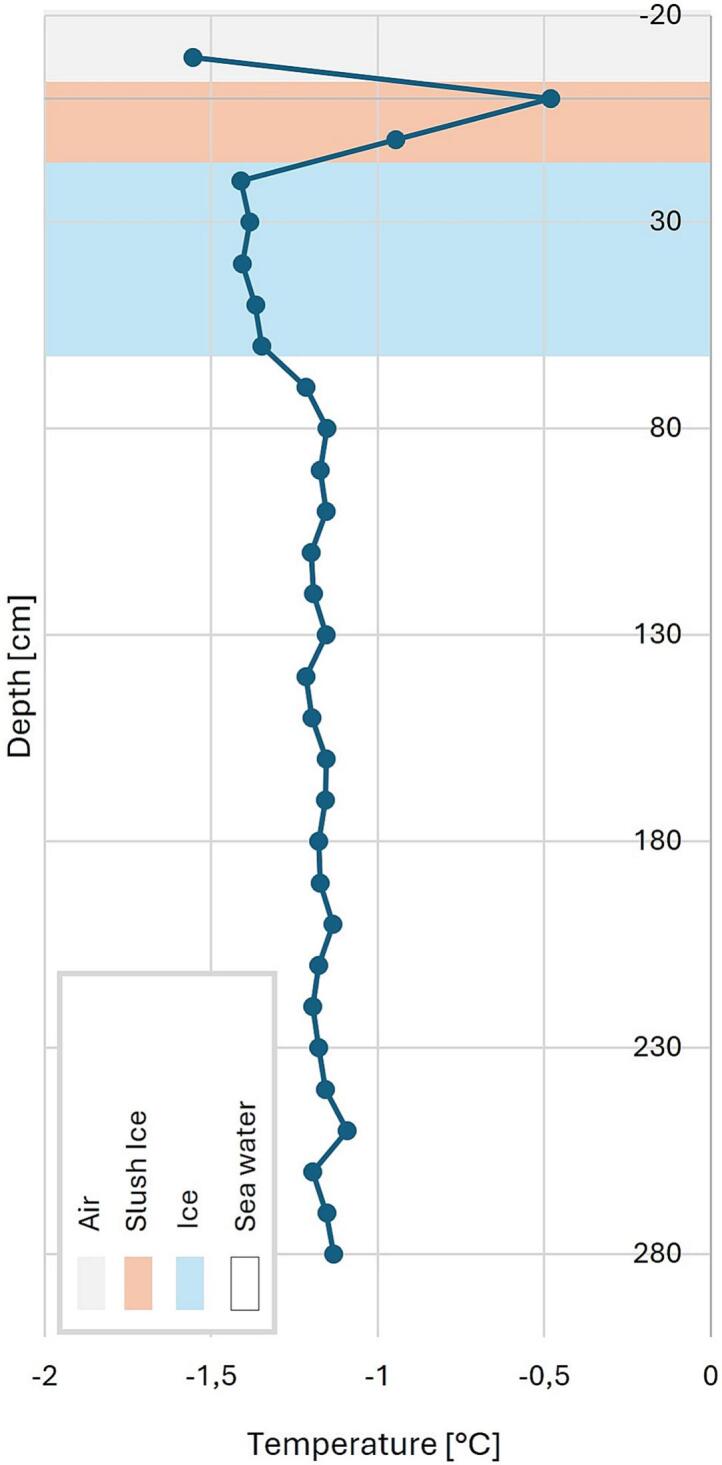
Test measurement in Nuuk. Vertical temperature profile measured and transmitted by the TS sensor system. The TSD was deployed by the robotic “Rover” through a drilled hole in the sea ice.

The TSD was tested in March 2024 in Nuuk (Fig. 11). The equipment was here tested with the focus of the functionality of the equipment. Starlink was used to establish a satellite-based internet connection. The Starlink dish and router was powered by a Honda EU20i generator [11]. A 5 cm hole was drilled with a Kovac Ice drill and the TS was lowered into the hole. Afterwards the DLB was placed over the hole. The data shown in Fig. 12 was achieved from here with the measurements successfully uploaded in real time.

At this location, it was not possible to leave the equipment and allow the hole to freeze up again.

### 7.2 Freezing test in the laboratory

**Fig. 13:**
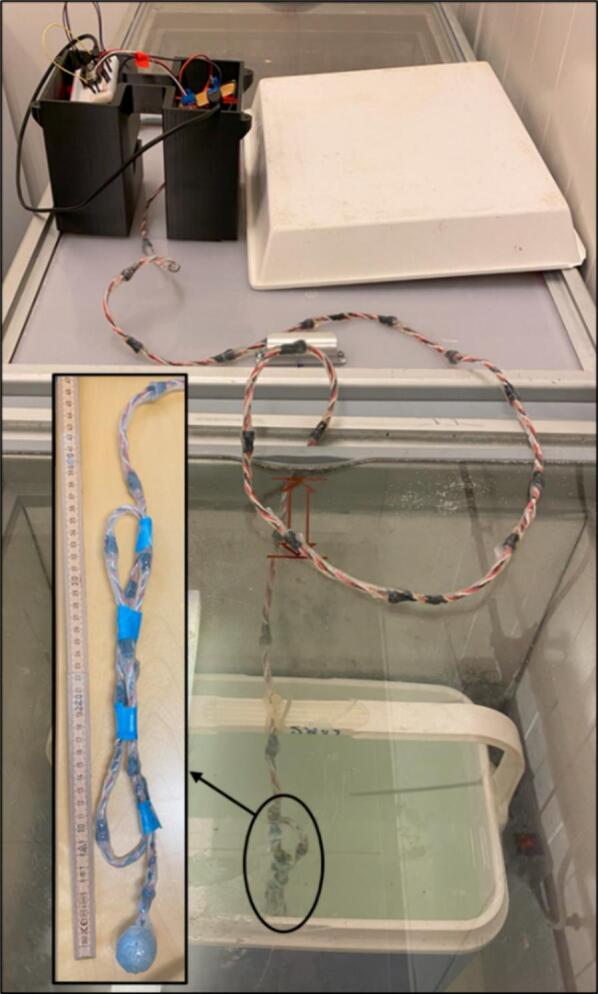
Test setup for freezing test in lab.

Additional validation was made in a freezer. Sea ice was grown in an insulated container holding 22 L of saline water with 33 g/litre NaCl. The DLB was connected to Wi-Fi through a mobile hotspot from a laptop. The TS was folded to gain a spatial resolution of approximately 3 cm using 8 sensors (Fig. 13). As sea water freezes from top and downwards, a heating element (5 W) was places in the bottom of the container to prevent sea ice from forming on the sides or bottom of the container.

**Fig. 14:**
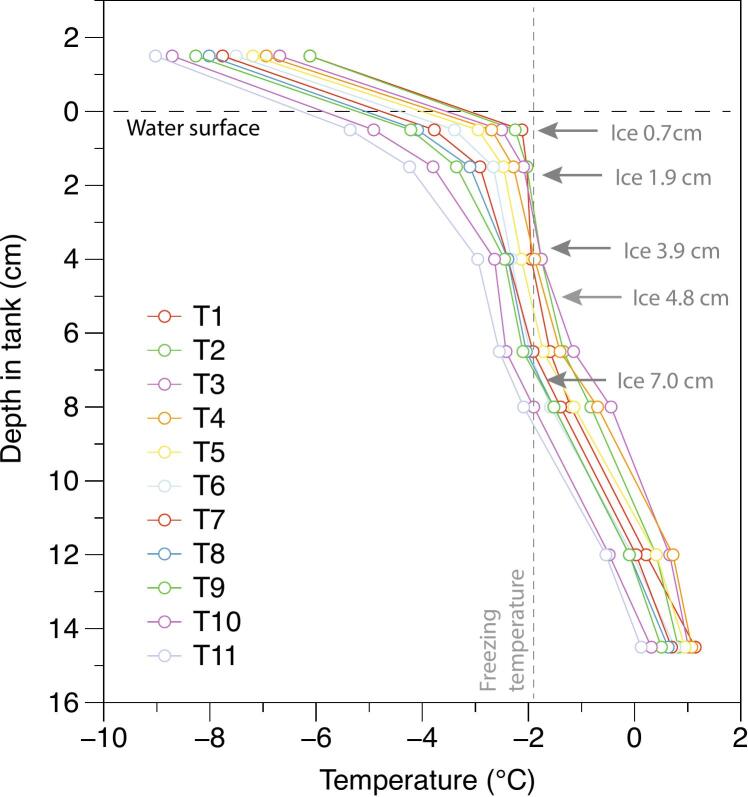
Measured temperature and sea ice thickness in the experimental laboratory setup. A vertical temperature profile is shown for each 6 h (T1 – T11) during the initial sea ice freezing.

The test was performed over 5 days. The results can be seen in Fig. 14 where the temperature gradually decreases, and sea ice are formed.

### 7.3 Endurance test in saline water

The TS has been exposed to saline water for 90 days. No changes in material properties, e.g. flexibility, lack of waterproofness and electrical conductivity, were observed during the testing period nor afterwards.

### 7.4 Capacities

- 1 year of operation with data collection every 30 min with inclusion of microcontroller sleeping process
- Temperature measurement resolution of 0.0625 ${\;}^{{}^{\circ}}$ C.
- Data collection interval: 30 min on default, however, it can be set to any.
- Deployable using a robot.

### 7.5 Improvements

The following improvements are recommended to be implemented to make it ready for the field.

1) Fill DLB with epoxy to protect the batteries and electronics, preserving their functionality when the ice melts and the unit submerges.
2) Attach a buoy to the DLB for easy localisation and retrieval later using echo signals.
3) To ensure a long battery life, it is recommended to include a sleeping process of the microcontroller between each data collection.
4) Add a GPS module and get the TSD position and real time clock this way.

## CRediT authorship contribution statement

**Lasse Alexander Nissen Pedersen:** Writing – review & editing, Writing – original draft, Validation, Software, Methodology, Investigation, Conceptualization. **Jeppe Don:** Writing – review & editing, Writing – original draft, Validation, Software, Methodology, Investigation, Conceptualization. **Claus Melvad:** Writing – review & editing, Validation, Supervision, Project administration, Methodology, Investigation, Conceptualization. **Søren Rysgaard:** Writing – review & editing, Writing – original draft, Supervision, Investigation, Funding acquisition, Conceptualization.

## Funding

This work was supported by Aage V Jensens Foundations “Prototypetest af robot til klimaforskning i Grønland” [grant number 2023–0031] and the Danish National Research Foundation [grant number DNRF 185].

## Declaration of competing interest

The authors declare that they have no known competing financial interests or personal relationships that could have appeared to influence the work reported in this paper.
